# Health Tract, No. 39

**Published:** 1861-06

**Authors:** 


### HEALTH TRACT, NO. 39.

### PRESENCE OF MIND.

1. If a man faints, place him flat on his back and let him alone.

2. If any poison is swallowed, drink instantly half a glass of cool water with a heaping teaspoonful each of common salt and ground mustard stirred into it; this vomits as soon as it reaches the stomach; but for fear some of the poison may still remain, swallow the white of one or two raw eggs or drink a cup of strong coffee, these two being antidotes for a greater number of poisons than any dozen other articles known, with the advantage of their being always at hand; if not, a half-pint of sweet-oil, or lamp-oil, or "drippings," or melted butter or lard are good substitutes, especially if they vomit quickly.

3. The best thing to stop the bleeding of a moderate cut instantly, is to cover it profusely with cob-web, or flour and salt, half-and-half.

4. If the blood comes from a wound by jets or spirts, be spry, or the man will be dead in a few minutes, because an artery is severed; tie a handkerchief loosely around near the part *between the wound and the heart!!* put a stick between the handkerchief and the skin, twist it round until the blood ceases to flow, and keep it there until the doctor comes; if in a position where the handkerchief can not be used, press the thumb on a spot near the wound, *between* the wound and the heart; increase the pressure until the bleeding ceases, but do not lessen that pressure for an instant, until the physician arrives, so as to glue up the wound by the coägulation or hardening of the cooling blood.

5. If your clothing takes fire, slide the hands down the dress, keeping them as close to the body as possible, at the same time sinking to the floor by bending the knees; this has a smothering effect on the flames; if not extinguished, or a great headway is gotten, lie down on the floor, roll over and over, or better, envelop yourself in a carpet, rug, bed-cloth, or any garment you can get hold of, always preferring woolen.

6. If a man asks you to go his security, say, "No," and run; otherwise you may be enslaved for life, or your wife and children may spend a weary existence, in want, sickness, and beggary.

7. If you find yourself in possession of a counterfeit note or coin, throw it in the fire on the instant; otherwise you may be tempted to pass it, and may pass it, to feel mean therefor, as long as you live, then it may pass into some man's hands as mean as yourself, with a new perpetration of iniquity, the loss to fall eventually on some poor struggling widow, whose "all" it may be.

8. Never laugh at the mishaps of any fellow mortal.

9. The very instant you perceive yourself in a passion shut your mouth; this is one among the best precepts outside of inspiration.

10. The man who always exacts the last cent, is always a mean man; there is no "evacuant" in all the "Materia Medica" efficient enough to "purge" him of his debasement; he is beyond druggery.

11. Never affect to be "plain" or "blunt;" these are the synonyms of brutality and boorishness; such persons are constantly inflicting wounds which neither time nor medicine can ever heal.

12. Never be witty at another's expense; true generosity never dwelt in such a heart; it only wants the opportunity to become a cheat or a rogue.

13. If the body is tired, rest; if the brain is tired, sleep.

14. If the bowels are loose, lie down in a warm bed, remain there, and eat nothing until you are well.

15. If an action of the bowels does not occur at the usual hour, eat not an atom until they do act, at least for thirty-six hours; meanwhile drink largely of cold water or hot teas, and exercise in the open air to the extent of a gentle perspiration, and keep this up until things are righted; this one suggestion, if practiced, would save myriads of lives every year, both in city and country.

16. The three best medicines in the world are warmth, abstinence, and repose.

